# The Dutch COVID-19 Contact Tracing App (the CoronaMelder): Usability Study

**DOI:** 10.2196/27882

**Published:** 2021-03-26

**Authors:** Britt Elise Bente, Jan Willem Jaap Roderick van 't Klooster, Maud Annemarie Schreijer, Lea Berkemeier, Joris Elmar van Gend, Peter Jan Hendrik Slijkhuis, Saskia Marion Kelders, Julia Elisabeth Wilhelmina Cornelia van Gemert-Pijnen

**Affiliations:** 1 Centre for eHealth and Wellbeing Research Department of Psychology, Health and Technology University of Twente Enschede Netherlands; 2 Behavioural Management and Social Sciences Lab Faculty of Behavioral, Management and Social Sciences University of Twente Enschede Netherlands; 3 Optentia Research Focus Area North West University Vanderbijlpark South Africa

**Keywords:** usability testing, user evaluation, user experience, contact tracing apps, CoronaMelder, COVID-19, pandemic, mobile apps, mHealth, public health

## Abstract

**Background:**

Adoption and evaluation of contact tracing tools based on information and communications technology may expand the reach and efficacy of traditional contact tracing methods in fighting COVID-19. The Dutch Ministry of Health, Welfare and Sports initiated and developed CoronaMelder, a COVID-19 contact tracing app. This app is based on a Google/Apple Exposure Notification approach and aims to combat the spread of the coronavirus among individuals by notifying those who are at increased risk of infection due to proximity to someone who later tests positive for COVID-19. The app should support traditional contact tracing by faster tracing and greater reach compared to regular contact tracing procedures.

**Objective:**

The main goal of this study is to investigate whether the CoronaMelder is able to support traditional contact tracing employed by public health authorities. To achieve this, usability tests were conducted to answer the following question: is the CoronaMelder user-friendly, understandable, reliable and credible, and inclusive?

**Methods:**

Participants (N=44) of different backgrounds were recruited: youth with varying educational levels, youth with an intellectual disability, migrants, adults (aged 40-64 years), and older adults (aged >65 years) via convenience sampling in the region of Twente in the Netherlands. The app was evaluated with scenario-based, think-aloud usability tests and additional interviews. Findings were recorded via voice recordings, observation notes, and the Dutch User Experience Questionnaire, and some participants wore eye trackers to measure gaze behavior.

**Results:**

Our results showed that the app is easy to use, although problems occurred with understandability and accessibility. Older adults and youth with a lower education level did not understand why or under what circumstances they would receive notifications, why they must share their key (ie, their assigned identifier), and what happens after sharing. In particular, youth in the lower-education category did not trust or understand Bluetooth signals, or comprehend timing and follow-up activities after a risk exposure notification. Older adults had difficulties multitasking (speaking with a public health worker and simultaneously sharing the key in the app). Public health authorities appeared to be unprepared to receive support from the app during traditional contact tracing because their telephone conversation protocol lacks guidance, explanation, and empathy.

**Conclusions:**

The study indicated that the CoronaMelder app is easy to use, but participants experienced misunderstandings about its functioning. The perceived lack of clarity led to misconceptions about the app, mostly regarding its usefulness and privacy-preserving mechanisms. Tailored and targeted communication through, for example, public campaigns or social media, is necessary to provide correct information about the app to residents in the Netherlands. Additionally, the app should be presented as part of the national coronavirus measures instead of as a stand-alone app offered to the public. Public health workers should be trained to effectively and empathetically instruct users on how to use the CoronaMelder app.

## Introduction

After the World Health Organization officially declared the COVID-19 outbreak as a pandemic, countries all over the world were urged to implement strict interventions in order to limit viral spread and to prevent overload of health care systems [1]. The key essentials of these interventions focus on reducing the risk of COVID-19 transmission and consist of a package of instruments that are implemented worldwide and are based on responses to earlier pandemics. They include behavioral measures (social distancing, handwashing, personal protective equipment), adequate resources (personnel and materials for massive-scale testing, contact tracing and supported isolation), monitoring symptoms (contact tracing of possibly infected persons), and the use of digital tools [2,3].

In traditional contact tracing approaches, public health authorities (PHA) follow protocols that aim “to interrupt transmission chains by ensuring that persons who have been in contact with an infected individual are notified that they are at increased risk of infection and how to take action to prevent passing the infection to others” [2]. This is important because although the coronavirus incubation period ranges between 1 and 14 days [4], an infected individual can transmit the virus up to 48 hours before the onset of symptoms [5]. In addition, some infected individuals do not develop symptoms but are still infectious [6]. According to the contact tracing protocol, PHA (1) contact positive-tested individuals, (2) advise them about measures, (3) identify together with them how or by whom they were infected, (4) list and contact all persons they have been in contact with, and (5) arrange for individuals’ contacts to be tested [7]. Despite being successful, traditional contact tracing by public health staff is labor-intensive, slow, and error-prone because people do not remember all their contacts [8,9]. Hence, the European Centre for Disease Prevention and Control has recommended the usage of digital tools, such as mobile tracing apps, to enhance and optimize traditional contact tracing [2].

Contact tracing apps could potentially provide several benefits as they (1) do not rely on the memory of the user (reminding users who they have had contact with), (2) allow contacts unknown to the user to be notified, (3) can speed up and enhance the tracing process, and (4) may facilitate further follow-up of contacts by PHA [2,6]. However, there are also some limitations in using these apps—not everyone has a smartphone or is able to carry their phone with them at all times; older smartphones or operating systems may not support newer apps (eg, newly developed apps can only operate on smartphones with operating systems iOS 13.5 or Android version 6, or later); the tracing technology inherently produces false positives and false negatives; and there are privacy concerns [2]. Furthermore, not everyone will be capable of or willing to use these apps (eg, older adults or vulnerable populations) [10]. These apps may complement but can never replace conventional contact tracing systems coordinated by PHA [10].

The Dutch Ministry of Health, Welfare and Sports (VWS) created conditions for the implementation of such an app. These conditions are listed in a *Plan of Requirements* [11] and mandate that the app should (1) be anonymous and voluntary to use; (2) be developed as open source (co-designed in an open Figma design platform); (3) notify users when they are at increased risk; (4) be in line with Guidelines for Infection Control [12]; (5) operate in addition to manual contact tracing (individuals should not receive help through the app that they would not receive without the app); (6) be inclusive (aimed at the largest possible relevant target group through explicit attention to language, literacy, and general/digital limitations); (7) aim to prevent reporting of false positives; and (8) involve international cooperation (eg, the app should be available on all phones operating on iOS and Android systems; connections between app users are made via Bluetooth; protection of privacy should be guaranteed [the app should be in line with common security standards and Web Content Accessibility guidelines, and a Data Privacy Impact Assessment should be performed]; and calculation of risks [distance, duration, and date of exposure] should be performed by the Google/Apple Exposure Notification framework [13]).

Contact tracing apps from other countries were examined by experts [14] to evaluate if these apps could also be implemented in the Netherlands. However, none of the evaluated apps met the above-mentioned criteria. The VWS therefore decided to develop a COVID-19 contact tracing app using the Google/Apple Exposure Notification framework that would be interoperable (to facilitate cross-border use): the CoronaMelder app. A development team, supported by an advisory committee and four task forces, was assigned to develop and test the CoronaMelder. The design of the app followed a privacy-by-design approach to minimize privacy invasion. During the development of the CoronaMelder, the app was tested with a variety of end users in field tests [15], think-aloud usability tests, practical tests, and ethical tests [16]. Additionally, the digital security of the app was tested using penetration tests and data privacy impact assessments. The findings of these tests led to continuous evaluation of the development and implementation of the CoronaMelder [16].

This paper focuses on the added value of the CoronaMelder app to support contact tracing. Think-aloud usability tests were conducted from June 29 to July 3, 2020, in the selected test region of Twente in the Netherlands. Twente was chosen for the current study because of its willingness to participate, available expertise, and the coronavirus-proof test infrastructure of the University of Twente. The usability tests aimed to evaluate the user-friendliness, understandability, reliability and credibility, inclusiveness, and user experience of the CoronaMelder. These criteria were chosen because the CoronaMelder could only support traditional contact tracing by PHA if the criteria are satisfied. As the app is to be used by the public, it needs to be understandable and usable by users varying in terms of digital literacy, educational background, and ethnicity. To this end, various target groups were involved in the usability tests. The findings of this study will contribute improvements in the design of the app and support the VWS in its decision whether to launch the app. This paper aims to answer the following question: is the CoronaMelder user-friendly, understandable, reliable and credible, and inclusive?

## Methods

### Setting

The study consisted of scenario-based usability tests with additional interview questions and the Dutch User Experience Questionnaire (UEQ-Dutch) [17]. The usability tests were conducted using a scenario-based, think-aloud method [18], captured by researcher observations and voice recordings, and took place from June 29 to July 3, 2020. A beta version of the app was evaluated using test iOS (version 0.1, build 172) and Android (version 0.3.1, build 107) phones. Mock-ups (Figma, version 0.7.1) were used for the scenarios that could not be tested in the beta version due to the current stage of development (ie, being able to download the app from the App Store). The study was conducted at the University of Twente’s DesignLab and in the Experivan mobile lab [19], which was used to visit participants with an intellectual disability. The BMS Lab protocol for coronavirus-safe research on humans has been approved by the Executive Board of the University of Twente. Hands and equipment were disinfected before and after the tests, and national measures were followed (per the National Institute for Public Health and the Environment [RIVM] [20]).

### Participants

Participants were recruited using convenience sampling in Twente.

To test whether the app is inclusive, participants from the following target groups were included:

- Youth (<21 years) with a lower level of education (n=14); this included primary education and prevocational secondary education;
- Youth (<21 years) with a higher level of education (n=5); this included senior general secondary education, preuniversity senior secondary vocational education, higher vocational education, and university education;
- Youth with an intellectual disability (n=4);
- Migrants (n=2);
- Adults (40-64 years) (n=5);
- Older adults (>65 years) (n=14).

### Ethics Approval and Consent to Participate

The study was approved by the university’s Ethical Committee (BCE200953).

Participants were informed of the voluntary nature of their participation and confidentiality was guaranteed. All participants signed an informed consent, and a parent or guardian signed the informed consent for underaged participants.

### Procedure

**Figure 1:**
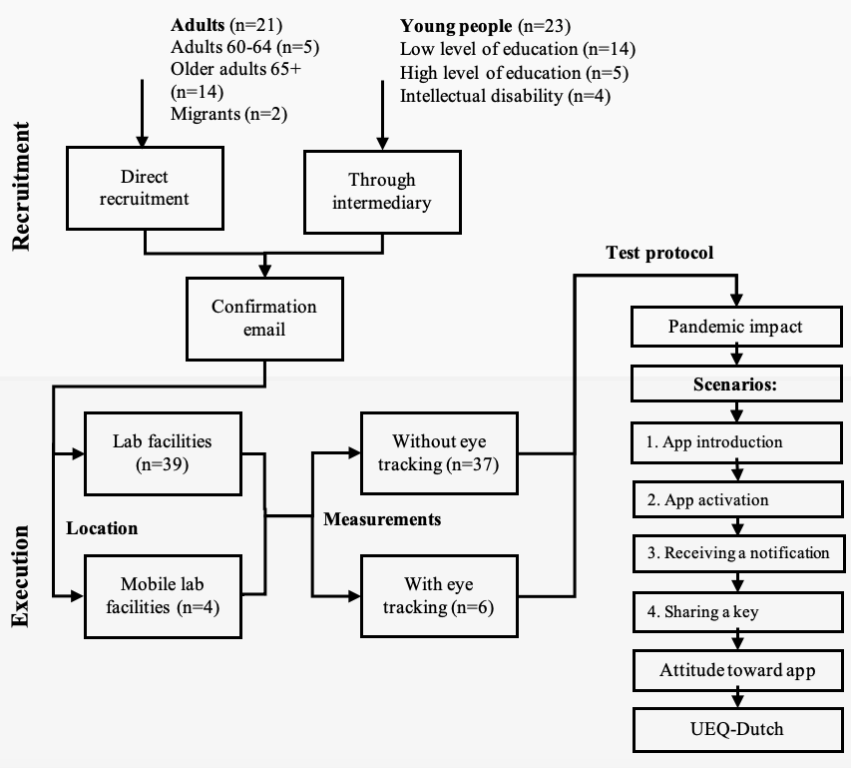
Flowchart of study recruitment and procedure. The top part visualizes the recruitment methods, through both direct (n=21) and indirect (via intermediaries/representatives, n=23) channels. Included participants were tested in a stationary and mobile lab, with or without additional eye tracking, following the test protocol depicted on the right. UEQ-Dutch: Dutch User Experience Questionnaire.

Adults (aged 40-64 years), older adults (aged >65 years), and migrants were contacted by the researchers by telephone. In this call, an explanation was provided about the nature and purpose of the study and an appointment was scheduled for the test. Recruitment of the younger participants was arranged through an intermediary (eg, school principal, mentor). The parents or guardians of minors were contacted by the intermediary via telephone and asked for their permission to let their children participate in the study. After the call with the researcher, all participants (or intermediaries) received email confirmation of the appointment, which also included additional information on participation. Figure 1 provides an overview of the procedure for participants.

The usability tests were conducted individually and in pairs in the case of youth in the lower-education category since research shows that minors are more capable of identifying a larger number of problems and details while working in pairs [21]. Before the test, the nature and purpose of the study were explained again, and permission for participation and audio recordings was given by signing the informed consent form. Parents or guardians had to sign the informed consent for minor youth. After providing additional consent, 6 participants additionally wore Tobii eye-tracker glasses (Tobii Gaming) for gaze analysis. An eye tracker was used due to people’s strong urge to move their eyes, so that the fovea is pointed at whatever stimulus they are currently thinking about or processing. This is referred to as the so-called eye-mind link [22-24]. This theory makes eye tracking a reliable tool for exploring visual attention. These results are discussed elsewhere [25] and are outside of the scope of this paper.

The test protocol (Multimedia Appendix 1) started with general questions about the impact of the pandemic on the participants’ lives and about what they had already heard about contact tracing apps for COVID-19. Thereafter, each participant actioned four scenarios on the app, which represented actual use of the app (1-hour test) while simultaneously thinking aloud. Before the usability test started, participants could choose between an iOS and Android test smartphone, per their preference. The four scenarios were:

1. Introduction to the app: in this scenario, the app was shown in the App Store, and additional information about the app could be read. Researchers focused on whether participants understood how to download the app and where they could find additional information, as well as whether they read the information.
2. Onboarding and activation of the app: in this scenario, the app’s operation was explained through onboarding steps, in which participants learned about the content of the app and confirmed the right settings for app use (allow the app to use Bluetooth and to send notifications). After onboarding was completed, the app was activated and participants had the opportunity to explore the app independently. Researchers focused on whether the explanation of the app’s function was clear, how participants acted, and whether they understood the permissions they consented to.
3. Receiving notifications: in this scenario, participants receive a notification from the app about their increased risk of infection because they have been in close contact with an individual who has tested positive for COVID-19. Researchers focused on whether participants understood what a notification entailed, whether it was clear to them why they received a notification, and what an increased risk status was based on. It was also examined whether it was clear to the participants what actions they should take after receiving a notification.
4. Sharing keys (telephone conversation with PHA): in this scenario, participants were asked to imagine they had recently been tested for COVID-19. During the scenario, participants received a phone call from a PHA worker, informing them of a positive test result. The PHA worker followed the Dutch Gemeentelijke Gezondheidsdienst (Regional Public Health Services) telephone script (Multimedia Appendix 2), in which the participant was asked about their symptoms and received help with sharing their key (ie, their assigned identifier). First, the participant had to provide the key to the PHA worker by phone. Next, the participant had to click a button on the app interface to share the key with other app users (to warn the people they were in contact with).

After completing the scenarios, closing interview questions were asked about the participants’ attitude toward the app and their willingness to use it. Additionally, a general questionnaire was administered (Multimedia Appendix 3), collecting data on gender, age, highest completed level of education, physical limitations in using apps, and a self-reported digital skills assessment, combined with the UEQ-Dutch. Within the UEQ-Dutch, participants had to assess different characteristics of the app (eg, whether the app is easy to use, visually attractive, supportive, and reliable) on a 7-point Likert scale. Researchers focused on whether the steps to be completed on the app were clear and easy to follow, and whether participants understood the utility and consequences of sharing their key. Differences between target groups were explored to investigate whether the app is inclusive. Whether the conversation with the PHA worker matched the steps that must be completed in the app was also examined.

### Data Analysis

The recordings of the usability tests were pseudonymized (by BB) and stored on a data server at the University of Twente and were only accessible to the researchers involved. These storage and associated processes were certified according to ISO/IEC (International Organization for Standardization/International Electrotechnical Commission) 27001 and NEN 7510 standards. Recordings of 3 participants (1 youth with a higher educational level and 2 youths with an intellectual disability) were not stored correctly due to technical errors, so these participants were excluded from the study. Recordings were transcribed verbatim (by MS, LB, JG), and all transcripts were analyzed (by BB and MS) to identify fragments on user-friendliness, understandability, reliability and credibility, and inclusiveness. Relevant fragments were labeled with the main codes “user-friendliness,” “understandability,” “reliability and credibility,” and “inclusiveness” in Microsoft Excel. Next, the fragments within the main codes were analyzed axially to link fragments to each other and create new subcodes within each main code. Two researchers (BB and MS) coded 6 transcripts together to determine coding agreements. BB and MS each coded half of the other transcripts while considering the coding agreements. The coding scheme was revised several times by both researchers, and fragments were reread and recoded if necessary. 

Descriptive statistics were generated for the general questionnaire using SPSS, version 20 (IBM Corp) [26]. The outcomes of the UEQ-Dutch were analyzed within the data analysis tool [27] offered by UEQ. This tool is benchmarked every year by UEQ to keep the validity of the tools as high as possible. The data gathered from the UEQ-Dutch questionnaire were input into the tool, which resulted in descriptive statistics and graphical representations of the gathered UEQ-Dutch data.

### Availability of Data and Materials

The transcribed data are not publicly available due to privacy restrictions but are available from the corresponding author upon reasonable request. All screenshots of the CoronaMelder app are online, available via Figma.

## Results

This section discusses participant demographics as well as the user-friendliness, understandability, reliability and credibility, and inclusiveness results of the CoronaMelder app.

### Participant Characteristics

In total, data from 44 participants were included in this study. The sample comprised 31 males (70.5%). The mean age was 40 years, ranging from 13 to 79 years, and 26 (59%) participants reported having completed a higher education level. The majority indicated not having physical limitations to using apps in general. Nearly all rated their digital skills to be between “not handy, not clumsy” and “very handy.” All participant characteristics are listed in detail in Table 1.

The following sections will focus on the research question: is the CoronaMelder user-friendly, understandable, reliable and credible, and inclusive? Table 2 presents an overview of how many arguments were mentioned by participants per theme, indicating their positive or negative opinion, or whether they understood the items related to the theme. The majority of the participants were positive about the user-friendliness, reliability, and credibility of the app. Participants from all target groups indicated more negative comments than positive ones regarding the understandability of the working mechanism of the CoronaMelder.

Multimedia Appendix 4 provides an overview of the number of positive and negative comments per topic, per target group.

**Table 1:** Demographic data of participants (N=44).

Characteristic			Value
Age (years), mean (range)			40 (13-79)
**Gender, n (%)**			
	Male	31 (70.5)	
	Female	13 (29.5)	
**Highest completed education level, n (%)**			
	None or primary education	12 (27.3)	
	Preparatory secondary vocational education (practical)	3 (6.8)	
	Preparatory secondary vocational education (theoretical)	3 (6.8)	
	Secondary vocational education	0 (0)	
	General secondary education/secondary university education	6 (13.6)	
	Propedeutic (higher professional education or scientific education)	4 (9.1)	
	Bachelor’s degree (higher professional education or scientific education	4 (9.1)	
	Master’s or doctoral degree	12 (27.3)	
**Physical limitations in using apps in general, n (%)**			
	I have trouble reading	2 (4.5)	
	I am dyslectic	1 (2.3)	
	I am visually impaired	0 (0)	
	I have a motor disability	0 (0)	
	I am hard of hearing	1 (2.3)	
	I have limited digital skills	2 (4.5)	
	Other (please specify)	1 (2.3)	
	None	36 (81.8)	
	Did not state	1 (2.3)	
**Self-reported digital skills assessment, n (%)**			
	Very handy	9 (20.5)	
	Handy	22 (50.0)	
	Not handy, not clumsy	12 (27.3)	
	Clumsy	1 (2.3)	
	Very clumsy	9 (20.5)	
	I do not know, no opinion	0 (0)	

**Table 2:** Number of participants who stated a positive or negative comment about the CoronaMelder per topic (user-friendliness, understandability, reliability and credibility, and inclusiveness). For understandability of the notification and key-sharing system, the table demonstrates how many participants understood how the CoronaMelder app worked.

Theme			Positive/understand, n (%)		Negative/did not understand, n (%)
**User-friendliness**					
	Layout	23 (53)		8 (18)	
	Navigation	33 (75)		9 (20)	
**Understandability**					
	Language	7 (16)		10 (23)	
	Receiving a notification	15 (34)		21 (48)	
	Sharing the key	15 (34)		19 (43)	
Reliability and credibility			19 (43)		6 (14)
Inclusiveness			5 (11)		9 (20)

### User-Friendliness

**Figure 2:**
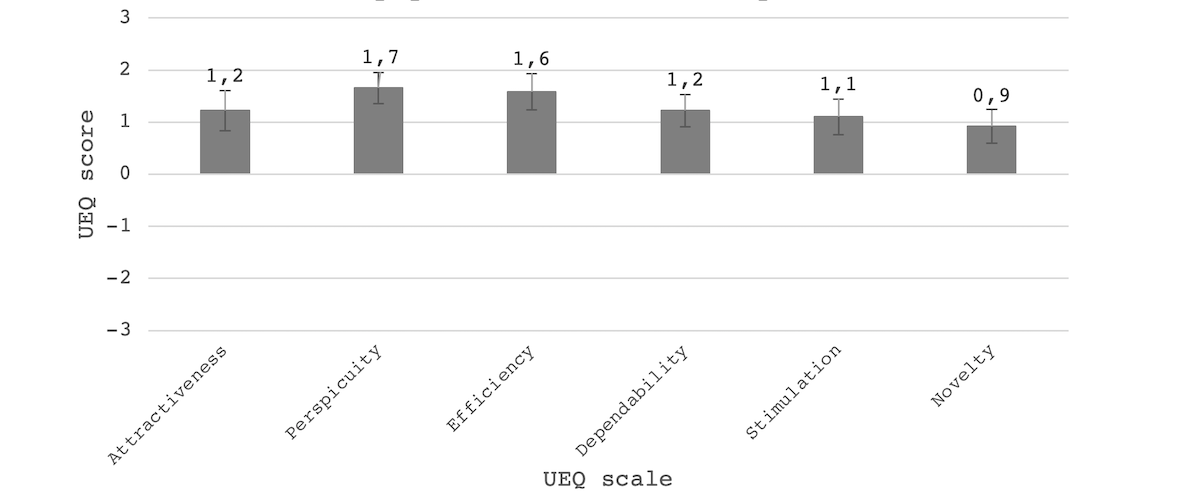
Boxplot with a mean score per User Experience Questionnaire (UEQ) scale for the total population, scoring from –3 (horribly bad) to +3 (extremely good). The error bars show confidence intervals.

User-friendliness was assessed with the UEQ-Dutch and during the interview. In Figure 2, the outcomes of the UEQ-Dutch for the entire population are displayed for each of the six assessed scales. The scales include *attractiveness* (an overall impression of how much users like or dislike the app), *perspicuity* (how easy it is for participants to become familiar with the app), *efficiency* (the ability of users to use the app as intended), *dependability* (whether the user feels in control of the interaction with the app), *stimulation* (whether the app is exciting and motivating to use), and *novelty* (whether the app catches the interest of the user).

Participants rated the CoronaMelder app between 0.9 and 1.7 for all scales, which represents a positive evaluation (values >0.8) [28]. For perspicuity and efficiency, the app was rated relatively high, indicating the CoronaMelder is easy to become familiar with and participants were able to use the app as intended. The CoronaMelder scored relatively low on novelty, indicating that it was not proficient in capturing user interest.

#### Layout

**Figure 3:**
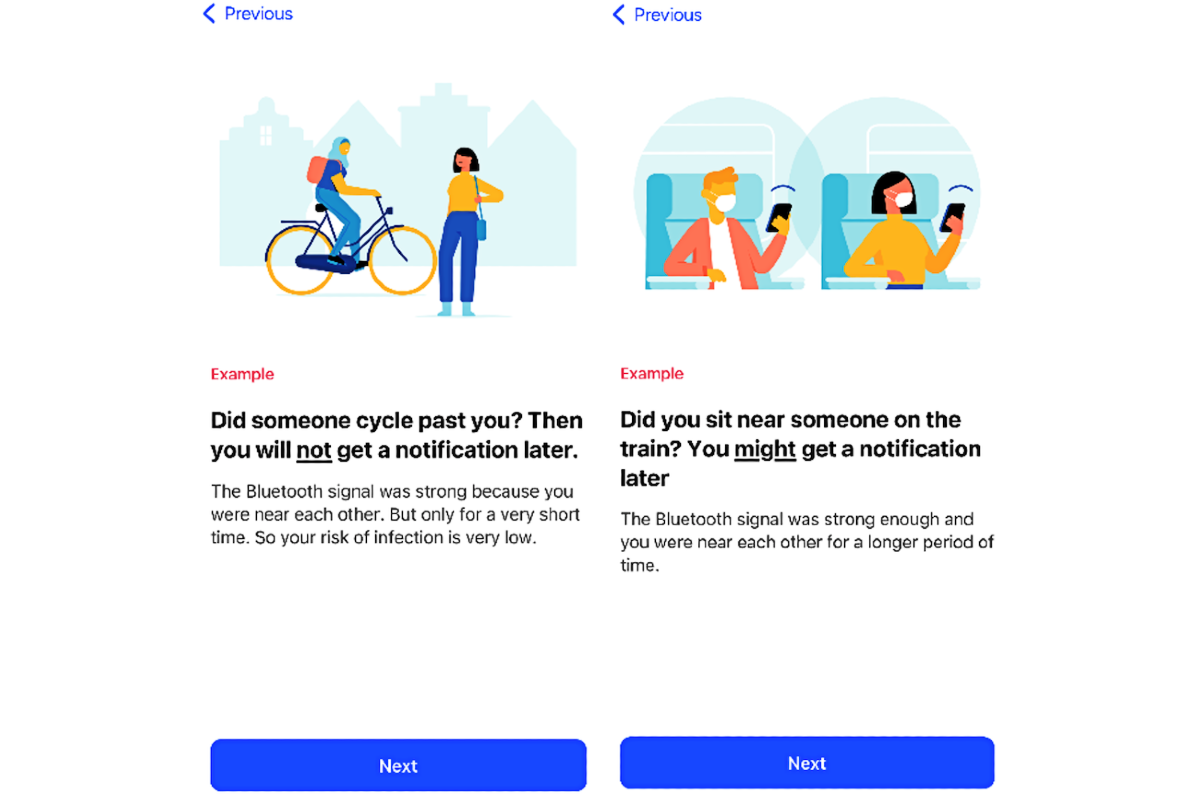
App screenshots viewed during onboarding: (left) an example of when users are not exposed to increased risk and (right) an example of when users might be exposed to increased risk.

The interviews showed that most of the participants liked the style of the app. The use of pictures within the app was appreciated, particularly by youth with an intellectual disability, who indicated having difficulties reading long texts. Representation and inclusiveness were achieved by displaying images of people from different cultures (Figure 3). The examples of when exposure leads to an increased risk (Figure 3) were commended since they helped participants better understand when they were exposed. Below is a sample response from participant 38, a youth with an intellectual disability:

Question: Based on what you’ve read so far, what things do you like and recognize as being important for you?

Participant: That it is explained in a simple way and that not too many words are getting used.

Question: And do you think that this is addressed here?

Participant: Yes.

One negative aspect included participants’ report of not reading long texts or only quickly scanning a text by reading the subheadings and words in bold to understand the most important information:

Well, I only read bold letters. I always want to be able to download an app quickly, so I am not going to read everything. The smaller, non-bold letters are then less important, I guess, so actually I skipped those.

Participant 2, adult

A few participants suggested that videos in which the information within the app is explained in more detail would be useful because not everyone likes to read, or some may not be able to read well. Additionally, using visualizations was recommended: youth with a lower educational level and those with an intellectual disability were not able to understand how the app worked by only reading the informational texts within the app:

It might have been useful to design a human with a mobile phone, standing with a group of people, one of whom appeared to be infected, and they all use the app. If there are 5 people and 1 have the virus, then if you make it visual how the app makes contact with other apps, you can explain what Bluetooth does. Because I don’t know if everyone knows what Bluetooth is.

Participant 20, adult

#### Navigation

**Figure 4:**
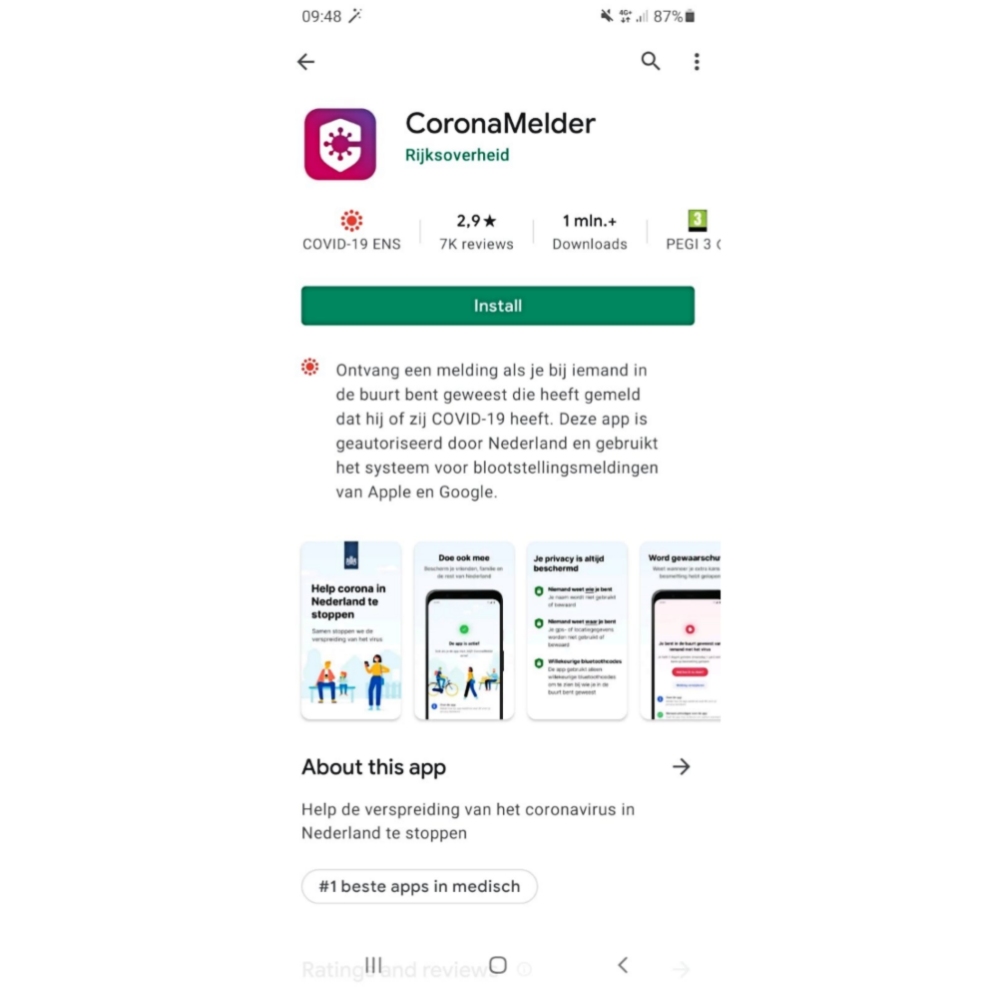
The CoronaMelder app as seen in the Google Play Store. The app can be installed by clicking the *Install* button. The text below this button reads: “Receive a notification if you have been in close contact with someone who later tested positive for COVID-19. This app is authorized for use in the Netherlands and uses the Google/Apple Exposure Notification system.” Additional information can be found in the *About this app* section. The text under this heading reads: “Help to stop the spread of the coronavirus in the Netherlands.”.

Most comments on navigation were positive. For example, participants considered the flow of information to be logical. Regardless, multiple issues with navigation were identified. First, it was unclear to participants whether an additional information page with more explanations about the app could be opened in the Google Play Store (Figure 4); hence, the page was not read by every participant. In particular, older participants did not know it was possible to consult additional information, although they reported wanting to read extra information.

Second, within the app, some buttons (eg, *Sharing the Key* and *Request a Corona Test*) were only visible after scrolling, which was not clear for some participants, who therefore could not find the buttons:

I didn’t know there was another fourth button […] Sometimes you think that what is visible here, is everything. Then I will not go scrolling automatically, but it could just be me.

Participant 27, adult

**Figure 5:**
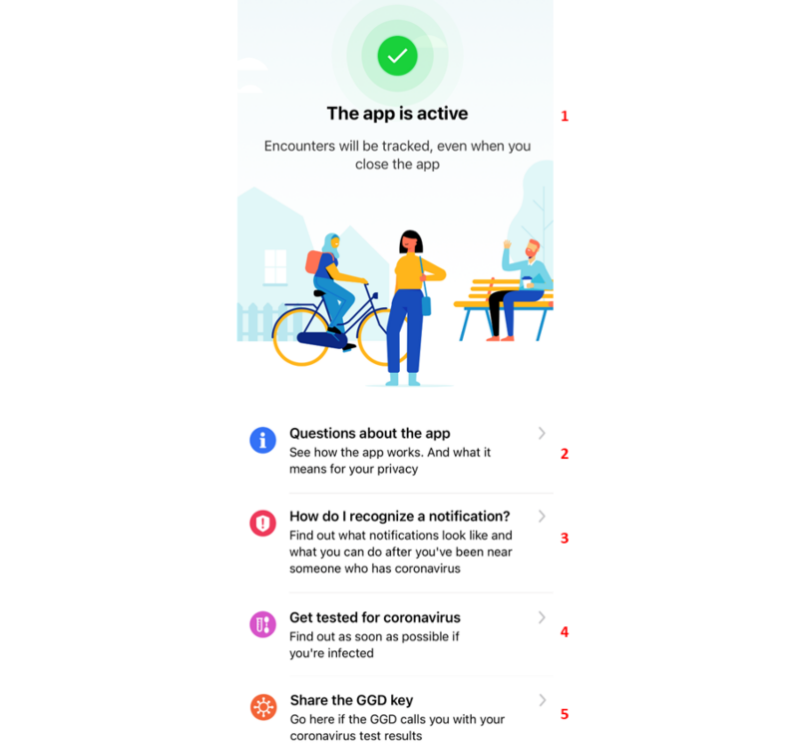
The app’s home screen, which appears after the user has finished the onboarding and activation of the app. At the top (1), the app explains its activation for use. Below this are several information pages on (2) how the app works (frequently asked questions), (3) what to do if one receives a notification, (4) how to request a COVID-19 test, and (5) how to share one’s key.

Third, the app did not provide clear expectations to the participants on what to do after activating the app. After activation, the home screen (Figure 5) is displayed, informing users that the app is activated and ready for use. Younger participants automatically closed the app, whereas older participants became disoriented and reported being unsure about what to do next:

You could also put that in the text ‘the app is active and if you are sufficiently informed you don’t have to do anything’, because people will ask ‘what now?’

Participant 8, adult

Positive remarks on navigation included the ease of onboarding and activating the app and the logical sequence flow of providing information about the app during onboarding (Multimedia Appendix 5). The majority of both older and younger participants indicated that they normally would click quickly through the screens and only read the information properly since they were participating in the study. The younger participants gave permission to receive notifications and use Bluetooth without delay, while the older participants thought carefully about granting permission:

Most of it is very logical. I now read the texts properly, but usually I would probably click and skip faster through it.

Participant 4, older adult

### Understandability

Participants from all target groups provided more negative comments than positive ones on the understandability of several topics. Understandability problems occurred due to inconsistency in the terms used and not reading information on how the app works. Most problems concerned receiving a notification from the app and sharing the key when tested positive to support contact tracing. The app was inconsistent in the ways it referred to the coronavirus (eg, “coronavirus,” “COVID-19,” and “corona”) and to the key that should be shared after testing positive (eg, “ID,” “code,” “control code”). Furthermore, a clear definition or explanation was lacking within the app about what it means to be exposed (“exposure”) or at “increased risk” of a COVID-19 infection. The texts also include technical English vocabulary, such as “ID,” “share,” “enable,” and “upload.” Older participants in particular did not know what those words meant and were confused by them:

Now suddenly some English words are used. Well, that is a problem for some people. You should not do that. Or you should provide both languages. But now you have people who get stuck around here […] this can confuse people.

Participant 4, older adult

#### Understandability of Receiving Notifications When Exposed to an Increased Risk

The app sends a notification to the phone, explaining that the user is at increased risk because on a certain date they were close for more than 10 minutes to another app user who later tested positive. Opening the notification brings the user to an information page in the app that explains what the user should do.

##### Being at Risk: When and How a Notification Will Be Received

The test showed that the majority of participants, but in particular youth in the lower-education category, did not understand under what circumstances they would receive a notification; for example, participants believed they would receive an alarm immediately after exposure:

But I am not quite sure how it works exactly, whether it’s anonymous or when you receive a notification… If I understand correctly, you will receive a notification if you have been with someone for more than 10 minutes, but then you do not know whether the person is infected or not? And if they have been tested, you will receive a notification that they were infected. Can I figure that out based on this? Well, I don’t think so.

Participant 32, older adult

Youth in the higher-education category, adults, and older adults also reported that the time between exposure and notification (within 14 days) is too long, although they can imagine why this is the case. A few participants even labeled the app as useless when they did not receive a notification immediately after being exposed because by the time they receive the notification, according to them, it is too late to take appropriate action:

It would be nice if, for example, someone has corona, then if you walk by, your phone will beep at once, like a message will be given […] Because after five days, it is already too late. If someone has the coronavirus and you immediately get a message, then you know, oh I must keep my distance.

Participant 14, youth, lower-education category

None of the participants, regardless of age and education, understood when they were at increased risk for possible infection. For example, they were not aware of how long and how close they must be to someone who turned out to be infected to receive an increased risk notification. Participants also appeared not to understand that the app only communicates with other apps, so they will only receive a notification if the infected person also uses the app. It was unclear to participants that exposure detection was based on Bluetooth connection between different smartphones and based on actually being exposed to the infected persons themselves. The exposure threshold level of 10 minutes was questioned by participants. Participants (across all target groups) believed that they can also be infected when they are in close proximity to someone for less than 10 minutes.

But 10 minutes … isn’t that quite long? Suppose he has coronavirus and I stand near him for 2 minutes and then leave […] 2 minutes is enough anyway.

Participant 15, youth, lower-education category

##### What to Do After Receiving a Notification

While youth with both lower and higher educational levels indicated that the app adequately explained what to do after they receive a notification, both adults and older adults mentioned that the app does not provide clear advice and even gives contradicting advice. For example, the app advises staying at home but also continuing daily life while being aware of symptoms.

But what I do want to know – and I miss that in here – is: what should I do now? I would like to know very specifically: what should I do? What options do I have? […] Or even more socially democratic: we recommend the following […] Suppose if you receive a notification, I want to know what now? What should I do? Then I don’t need to know about symptoms or about a corona test...

Participant 12, adult

Participants emphasized that the app should clarify how it notifies users, and what users are expected to do after receiving this notification. A few participants reported that they did not find the information about symptoms of the coronavirus and the possibility of requesting a test useful; they preferred to read advice on what they should do at that moment:

If you get a notification, 1) I want to know how I get this notification, 2) suppose I have received a notification, what now? What am I supposed to do? I don’t need to know about symptoms or about a corona test then.

Participant 12, adult

#### Understandability of Sharing a Key After Testing Positive

Various understandability issues were identified in the scenario of a positive test, as well as some positive remarks. The issues revolved around not understanding what the key is, where to find it, when to share it, and how to share it. For example, some participants mentioned they did not know what the key involved or what they were expected to do:

Well, I see now that it [the app] works through a key and I haven’t read about that anywhere yet. So, I don’t know what that key involves.

Participant 12, adult

Participants were also unaware that a PHA worker would call the participant to initiate key sharing:

Now they say ‘then the PHA worker will ask you in the telephone conversation to share the key from the app and then upload the keys of the telephones you’ve been in contact with.’ What are they talking about? Which key? I don’t know what key they are talking about.

Participant 12, adult

A few youths in the lower-education category or with an intellectual disability did not understand how the app knew that one had received a positive test result:

But how does the app know you have corona? Do you have to type that in the app?

Participant 15, youth, lower-education category

Participants expressed different opinions regarding the text about sharing the key. Youth in the lower education category appeared to easily share the key and did not want information about what was expected from them. A few adults mentioned that the symptoms of the coronavirus and the implemented measurements are repeated too often within the app. The texts were considered to be too long, and it was reported that an overload of information should be prevented. Adults in particular expressed their indignation about the choice to share one’s key; they mentioned that if people were not willing to share the key, they should not have downloaded the app:

If I have the app, wouldn’t that obligate me to share the key? I think you should share the key, that this choice option doesn’t have to be in there. Otherwise, I wouldn’t have to install the app. We try to help get this virus under control, together. Together, therefore, means that you have to share this information with others. So, I think that this choice to share or not is ridiculous.

Participant 5, adult

The steps participants must perform to share the key were clear to most youth and adults, although some older adults did not understand which process starts after they click on the *Share Your Code* button. It was unclear to them why they must point the key to the PHA worker first and whether they had to share the key afterward with other users by clicking on the button. Some thought they must send the key to their contacts via other communication channels:

My question is whether if it says ‘share codes’, whether this relates to the person I’ve been in contact with […] or whether codes are shared with the PHA, and the PHA then warns people I’ve been in contact with. That’s unclear to me.

Participant 18, older adult

Some youths in the lower-education category pointed out that when the key is mentioned to a PHA worker, the app is not anonymous anymore since the PHA worker knows which key belongs to which person. Below is a sample exchange between participant 13 (youth, lower-education category) and the interviewer:

Participant: Well, now they suddenly have my number at the PHA?

Interviewer: Yes, that’s right, the corona test is not anonymous. But sharing the key is.

Participant: But if I share my key with them, then it is not completely anonymous, right?

**Figure 6:**
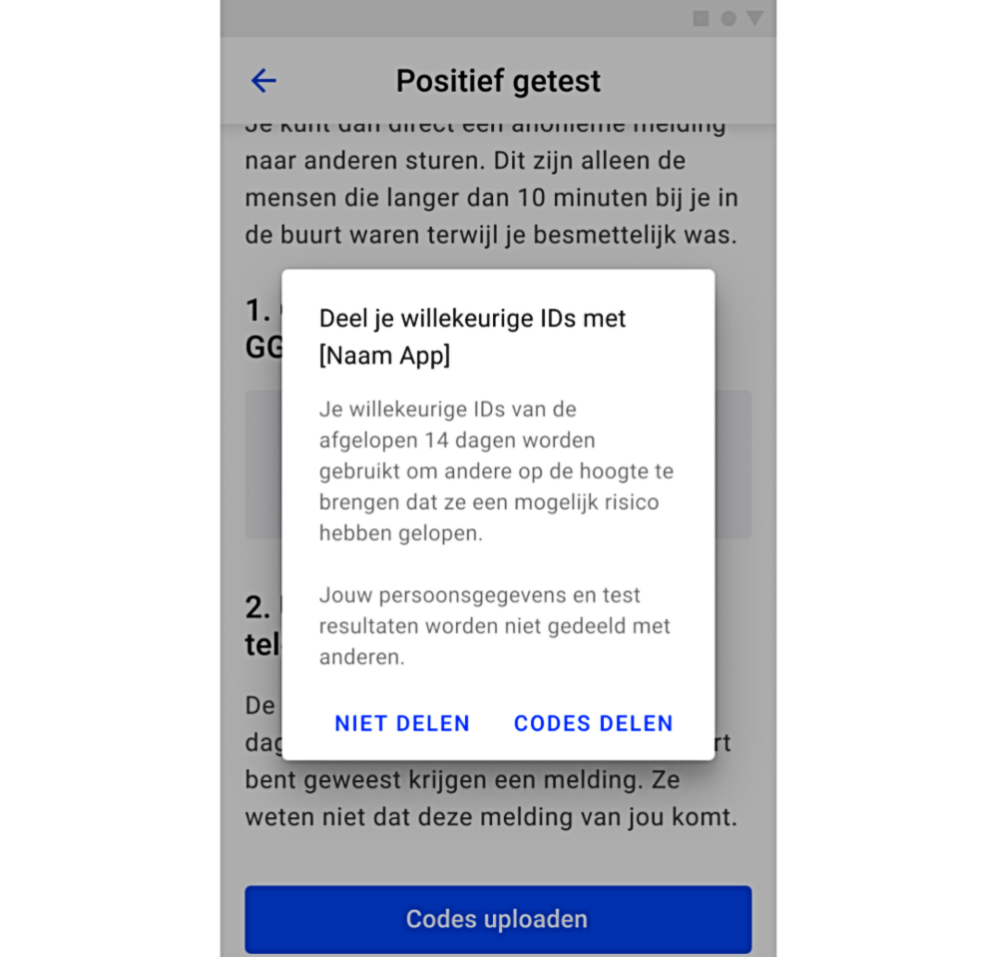
The notification that pops up after sharing the key, asking the user for permission to share their information and notify others after they click on *Share the Key*. The text reads: “Share your random IDs with [name of the app]. Your randomly generated IDs for the past 14 days will be used to notify others that they have been at increased risk. Your personal data and test results will not be shared with others.” The choice options are translated as “do not share” and “share codes.”.

In addition to this, some adults were irritated by the notification (Figure 6) that comes after sharing the key. They reported that one only used the app if one wanted to warn others and assessed the extra permission notification for sharing as unnecessary:

In my opinion, this is a strange choice. […] It was clearly emphasized at the beginning [onboarding] that the app is anonymous. I also think now, since I downloaded the app, I have to warn others. That’s not a choice, it’s a logical consequence of the fact that I installed the app. I specifically downloaded the app because it’s anonymized. This is just part of the deal. I have no idea who I’m sending it to [the key], I don’t know where I was [during the possible infection], I don’t know anything, but I do know that others will receive a signal just like I received a signal. Then I should no longer have the choice of sharing or not sharing.

Participant 5, adult

Participants, with the exception of older adults, considered the steps provided by the PHA worker for sharing the key on the app to be clear and easy to follow:

It was super easy for me. Even a young child can do this on their own.

Participant 2, adult

This is unclear, how to do that, because there is nothing, the screen says only ‘close’ to me […] Yes, because, what you said to me, I cannot carry out […] No, I only have to close the blue bar […] Maybe I can do that, maybe that will work […] No, I returned to the previous screen…

Participant 4, older adult

Participants did express that the simulated telephone conversation with the PHA worker lacks guided assistance, further explanations, and empathy. However, a telephone conversation was seen as a more personal approach, although youth with a lower educational level or with an intellectual disability preferred not to be called, and a few said they will not answer the phone if the PHA worker calls from a number with no calling ID. Additionally, too little attention was paid to the emotions that the message of testing positive can convey to participants. Participants suggested that PHA workers should make time to help them through the scenario, especially older adults, and should identify themselves to ensure reliability. Participants reported that the PHA worker should calmly go through the steps with the app users and not ask them to perform certain steps without providing an explanation:

I think it is pleasant if someone calls you and goes calmly through the app with you. That they don’t just say, well you have to do this and that, but that they really explain step by step what to do and where I have to click, in the app. That might be useful for older people or people who do not know or understand how the app works. I think that would be useful.

Participant 25, youth, higher-education category

### Reliability and Credibility

The app was assumed to be reliable because it was presented as a government app, and participants trust the VWS:

[Participant is reading additional information in the App Store] ‘Released by the Ministry of Health, Welfare and Sports’. Well, that seems confidential to me.

Participant 19, youth, lower-education category

**Figure 7:**
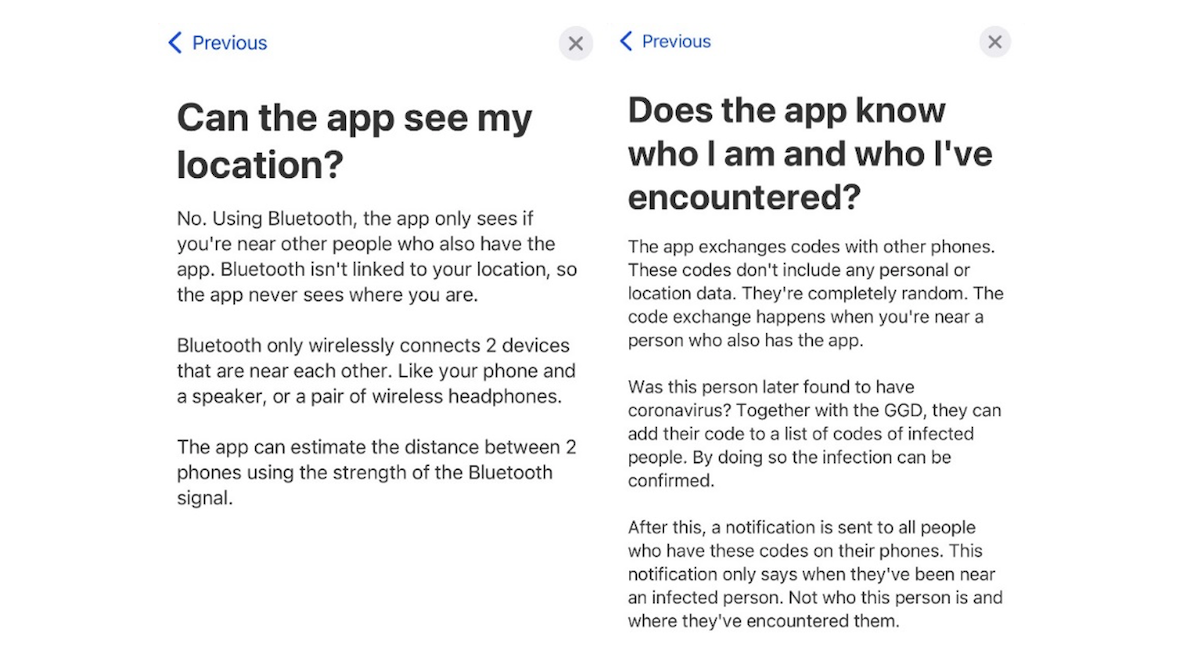
Screenshot of frequently asked questions, with responses (left) indicating that the app does not track users’ GPS and (right) how the app is anonymous.

The explanation about data storage and anonymity (Figure 7) earns trust, and adult participants in particular applauded the fact that the app does not require personal data.

I think it’s good that they clearly state what makes the app safe and anonymous, I think that’s strong […] Otherwise if these weren’t listed it would scare people off.

Participant 21, youth, higher-education category

Although adults assessed the app as trustworthy, youth in the lower-education group did not understand how the app guarantees privacy. Participants (from different target groups) believed that they would be tracked and that others would know their name and address when they reported a positive diagnosis. These misconceptions are not only caused by a lack of explanation or by participants not reading the information provided, but also because participants mistrust the use of Bluetooth—some participants may think it will still track their location and that the app will connect with people who are not directly in close proximity to them (eg, separated by a wall):

It says that the app knows via Bluetooth whether you were close to someone […] The app doesn’t know where you were and who you are. But that’s nonsense, it has to be. If you turn on Bluetooth, you immediately see where someone is […] That’s through the Apple satellite, same for the Samsung satellite. They can always track your phone, it doesn’t matter if you have turned it [GPS tracking] off. That’s why it’s nonsense, and they should add that. But well, if that is the case, if I already know that someone will use my location, I will immediately delete the app.

Participant 15, youth, lower-education category

Other participants were less doubtful about the use of Bluetooth, reporting that they thought the acquisition and storage of data was safe. Additionally, if privacy is guaranteed, multiple participants (mostly youth in the higher-education category and adults) mentioned their willingness to use the app:

Of course, in relation to privacy, you always check who monitors what data, but that will undoubtedly also be properly secured and your GPS location data will not be used or stored. That sounds safe, and I assume it is.

Participant 4, older adult

### Inclusiveness

Differences in inclusiveness were found across age groups. In general, participants from all target groups with the exception of older adults did not have any problems using the app. Among older adults, there was a dichotomy between those who had digital skills and those who had trouble finding specific information within the app, opening and closing specific screens, and attending a telephone call while simultaneously opening and using the app:

Turn on speaker, that will be interesting. I’m going to see if I can do that. Speaker, yes, I did it! I succeeded. Well, to the Corona app, let me ask, how do I get there? These are things I’m not handy with. I have to go to the app. It works on my own phone, but now it won’t. Close everything … no, I shouldn’t do that. Ah, this one. Yes, I’m in the Corona app now.

Participant 29, older adult

Older adults were able to perform the steps to share the key under the guidance of the PHA worker but had difficulty performing these steps while talking on their mobile phone. For example, older adults were not aware of how they could turn on the speaker or close the call screen and open the app. However, they signaled willingness to learn how to use the app:

Oh that’s difficult, then I have to make a phone call and look something up in the app. I don’t know how to do that. Normally I can’t even answer my phone when I’m doing something else on my phone.

Participant 1, older adult

Additionally, youth with an intellectual disability appeared to lean on the researcher while conducting the test. They were in doubt and asked for confirmation each time before clicking on a screen or button. They appeared to not know what they were doing or why they must do it. For example, while sharing the key, they followed the steps the PHA worker provided them, without seeming to understand what they were doing or what would happen next.

Regarding the language used in the app, participants reacted differently according to their educational or cultural background. For example, youth in the higher-education category and both adult and older adults thought the language was clear and easy to understand and that appropriate words were used to express the purpose of the app:

Of course not everyone can read properly, that can be a bottleneck. The information should actually be as simple as possible. I think it is easy to read, but I don’t know if that applies to everyone.

Participant 11, adult

Youth with a lower educational level, youth with an intellectual disability, and migrants reported that the words used were too difficult to understand and texts were too long. The latter indicated that the app should work in other languages, such as English and Arabic.

P1: It has really difficult words…P2: I agree, and difficult words are annoying to read

Paired participants 16.1 and 16.2, youth, lower-education category

Some words I don’t understand so well. It would be easier for me if I could choose another language.

Participant 41, migrant

## Discussion

### Principal Findings

This study aims to answer the research question: is the CoronaMelder user-friendly, understandable, reliable and credible, and inclusive? Based on the findings, we can conclude that the CoronaMelder is easy to use. The app was seen by most as a good initiative because it warns them about possible infections, protects them, and could help avoid a second viral wave. The app was considered reliable because it is an initiative from the government (VWS). After participants read the information in the App Store, they indicated understanding how the app operates, and many were curious to become familiar with the app and expressed their intention to download it. Several general reasons why participants were willing to use the app were indicated, such as protecting themselves and their loved ones, creating sufficient support for the app, and helping to get COVID-19 under control and ease nationwide measures. However, it appeared that essential parts of the app were not understood by the participants, such as the notification system, the sharing of the authorization key via PHA, or how the app guarantees privacy.

Doubts and fears were expressed regarding privacy, usefulness, and consequences of the CoronaMelder. Among the reasons for these negative attitudes were fewer positive arguments in the media and the number of false positives. Reasons not to use the CoronaMelder were expressed, such as perceiving the app as useless, thinking the coronavirus and corresponding measures were overrated, not wanting to be in quarantine (without confirmed risk), and limited phone memory or battery capacity. In terms of inclusiveness, it appears the CoronaMelder is not accessible to various target groups. Youth at a lower educational level or those with disabilities had difficulties using the app due to low literacy and language problems, and the older adults experienced difficulties related to limited digital skills.

Whether the app will be effective in supporting traditional contact tracing is a concern since the majority of participants did not understand how the app operates or why there is a delay between exposure/increased risk and receiving a notification. The app provides complex information and lacks explanations; therefore, users find it unclear what actions the app expects from them. This lack of clarity has led to misconceptions about the app in terms of operation, privacy, and usefulness, thus affecting participants’ willingness to use it. This also affects the adoption of the app and adherence. Additionally, the protocol of PHA workers lacks guidance, explanation, and empathy, revealing that PHA staff are unprepared to support users with the app to the fullest extent during the pandemic (eg, with key-sharing procedures) in addition to their other responsibilities.

### Comparison With Studies on Other COVID-19 Contact Tracing Apps

To the best of our knowledge, this is the first study to pretest the usability of the Dutch CoronaMelder app. Studies of other countries’ contact tracing apps that operate similarly to the CoronaMelder reported comparable findings on participants’ attitudes toward these kinds of apps. In a study by Horstmann et al [29] on the German Corona-Warn-App, participants indicated that there were no reasons not to use the app, that the benefits would outweigh the risks, and that they believed the app would contribute to slowing down the pandemic [29]. On the other hand, in studies of apps in Germany, Switzerland, and France, the most frequently mentioned reasons not to use the app were privacy concerns [29-35], doubts about usefulness [29,34,36], and lack of technical equipment (eg, not all smartphone operating systems can access the apps) [29,34,37]. In both of the studies on the Corona-Warn-App (Germany) [29] and the SwissCovid app (Switzerland) [34], as well as in a longitudinal survey about the Dutch CoronaMelder [38], privacy concerns appeared to be associated with a lack of trust in the national government (or in PHA).

In addition, the StopCovid app (France) showed low uptake [36,39] because the app appeared uninteresting and ineffective [36]. The same study also reported that 71% of their participants suggested that better communication strategies would increase uptake of the app. Our study stated that the reasons mentioned for not using the app were already expressed before contact tracing apps were launched. However, it was reported [29,40] that these concerns were still raised after app implementation, which indicates that public health campaigns promoting contact tracing apps were not able to eliminate these concerns.

### Lessons Learned and Recommendations

#### Adequate and Targeted Communication

The tests showed that lack of clarity led to misconceptions about the app, affecting participants’ willingness to use it. Communication about the app is therefore essential for acceptance [3,29,40]. Targeted and tailored group-specific communication should happen through channels such as public campaigns, animations, social media, and ambassadors or influencers [3,41]. Communication should be customized according to the aim of the app in relation to other national COVID-19 measures (eg, testing, quarantine, social distancing) [3] and in collaboration with PHA and family physicians.

Providing more explanations, plus emphasizing the advantages of the app in comparison to regular contact tracing, increases the likelihood that, when individuals download the app, they will know what to expect as well as what is expected of them [29,35]. Walrave et al [35] had already reported that the intention to adopt a contact tracing app increases if people know how to use the app. Hence, it would be important to have a helpdesk where people can ask questions, for example, about the purpose of contact tracing and how the CoronaMelder contributes to this, how the app operates abroad (ie, outside of the Netherlands), how anonymity is guaranteed, how data is stored, what the role of PHA will be, and who to approach in case of uncertainty or fear regarding possible risks of infection.

#### Embedment in Traditional Contact Tracing by PHA

Our study suggests that it is important for PHA workers to be well prepared in guiding users to share their keys in case of positive test results. It is important to consider the app not as a stand-alone tool but as part of the pandemic infrastructure [3,35,41] and to embed it within PHA workers’ workflow. Hence, it is crucial to provide access to tests, regardless of symptoms, but dependent on the contact date with an infected person; complete testing quickly and deliver tests results within 24 hours; clarify the scope of the app compared to other digital resources (eg, Dashboard, Thuisarts.nl) or apps to be developed; arrange international agreements about interoperability with contact tracing apps in other countries; facilitate effective and efficient interaction between PHA and the CoronaMelder; and evaluate the effects of the CoronaMelder on contact tracing, individuals’ behavior, and society.

PHA should coordinate how their health care workers can guide individuals through the steps of sharing the key with the app. PHA workers should be trained to properly and empathetically explain which steps people must follow on the app. After all, PHA workers are responsible for both conducting the conversation about the test result and instructing users on the app. This means that they should be well educated about the aim and operation of the app and about their task and role during the phone call. It is therefore recommended to examine ways PHA workers can proceed to effectively and empathetically interact with app users.

### Strengths and Limitations

The first strength of this study is its focus on participants of different backgrounds (age, education, etc) to test whether the CoronaMelder is accessible to all residents of the Netherlands. The second strength is the real-time pretesting of the key parts of the CoronaMelder to enable revisions before the definitive launch. The findings of the study were communicated with the software development team to exchange feedback on adjustments to the app and to revise the app during the test days. During development, minor adjustments in the app’s design were made, which means that participants who tested the app later in the study may have viewed some screens that were different to those tested by participants earlier, even though the essential parts of the app were the same. Based on the findings of this study, the VWS decided to launch the app. However, the definitive launch (October 10, 2020) was postponed due to changes in testing policy. The premise “test without symptoms,” which is an important driver for using the app, was changed due to a lack of testing capacity.

### Future Research

To fulfil the requirements of the CoronaMelder [11], the design of the app can be improved. The accessibility and understandability of the app should be customized to differences in literacy and digital skills. Think-aloud, real-world–based scenarios and eye tracking should be designed to involve end users with different literacy levels and digital skills to test use of the app in real time.

Evaluation of the CoronaMelder app should focus on the key essentials of the app to support early and better contact tracing. Data should therefore be collected on use and adherence regarding follow-up actions after a notification, sharing a key to inform PHA and other users (via the app), and going into isolation when testing positive. The privacy-by-design policy could complicate gaining insight into the added value of the CoronaMelder app since it hinders data collection. A critical view is needed on how to find a balance between user-centered design and the privacy-by-design policy.

Future studies should also focus on how communication campaigns can be best targeted and customized to reduce uncertainties and misconceptions, thereby improving the understanding of digital contact tracing apps as well as adoption and adherence. Overall, an adequate infrastructure (resources, personnel, capacities, etc) and powerful management tools are needed to implement the CoronaMelder and other digital tools to facilitate and optimize contact tracing to fight a pandemic. The COVID-19 pandemic has had an adverse global impact and requires an interdisciplinary approach. Future studies of the CoronaMelder app should consider the app not as a stand-alone device but as part of a coherent package of anti–COVID-19 measures to fight the pandemic, considering the impact on users, stakeholders, and testing and tracing procedures.

## Appendix

Multimedia Appendix 1 Test protocol.

Multimedia Appendix 2 PHA telephone script for positive test results.

Multimedia Appendix 3 The UEQ-Dutch questionnaire.

Multimedia Appendix 4 Number of participants per target group who stated a positive or negative comment about the CoronaMelder, per topic (user-friendliness, understandability, reliability and credibility, and inclusiveness). In the case of the understandability of the notification system and sharing of the key, the table shows how many participants understood how the CoronaMelder app worked.

Multimedia Appendix 5 Steps to onboard and activate the CoronaMelder app.

## Abbreviations

IEC: International Electrotechnical Commission
ISO: International Organization for Standardization
PHA: public health authorities
RIVM: National Institute for Public Health and the Environment
UEQ-Dutch: Dutch User Experience Questionnaire
VWS: Ministry of Health, Welfare and Sports
