# Supplementary material for: The Dutch COVID-19 Contact Tracing App (the CoronaMelder): Usability Study
Source: JMIR Form Res. 2021 Mar 26;5(3):e27882. doi: 10.2196/27882 (PMC8006901; doi:10.2196/27882)
Supplement: Multimedia Appendix 1 [file formative_v5i3e27882_app1.docx]

## Appendix 1 – Test protocol

**Protocol voor testers – COVID-19 app usability tests VERSIE 26 JUNI**

| Tijd | Taak | Doel |
| --- | --- | --- |
| *0* | Zorg voordat je de deelnemer ophaalt, dat je het volgende voorbereid hebt:  Voorbereiding gesprek:   - Zorg dat de opname apparatuur in de juiste positie staat en gereed is voor opname. - Zorg dat het informed consent en het ‘scoreformulier’ klaarliggen. Dit scoreformulier ga je tijdens de scenario’s invullen. Je vult hierin de meest belangrijke punten in, bijv. “deelnemer vindt tekst te lang; deelnemer snapt niet hoe hij/zij X moet doen”. Na afloop kun je samen met de deelnemer checken of het compleet is. Zorg dat je de opname tijd (zie GoPro) noteert bij de opmerkingen die je opschrijft. Bijv. persoon zegt op 13:49 dat hij de tekst te lang vindt, noteer dan: “deelnemer vindt tekst te lang, 13:49”. - Check of deze deelnemer tot groep A of B hoort bij scenario 4; leg flyer klaar indien scenario B.   Voorbereiding mobieltjes:   - Verwijder de app van het toestel en download hem opnieuw. Dit kan via de mail die Joris heeft gestuurd naar het toestel, hier zit een link in. Voor elke deelnemer moet de app verwijderd worden en weer opnieuw gedownload worden. - De mock-up (informatie in App Store): nog bepalen wat makkelijkste is 🡪 screenshots maken en onder foto’s laten zien? Zorg dat je zelf screenshots van de mock-ups achter de hand hebt op je laptop/tablet.   Corona maatregelen:   - Maak het test-mobieltje tussen de verschillende deelnemers door schoon met bijgeleverd desinfectiemateriaal. - Let ook op je eigen veiligheid, desinfecteer je handen en de spullen die je aanraakt tussendoor. | Ruimte klaarmaken voor test |
| *3 minuten* | Haal de deelnemer op. Vraag de deelnemer zich voor te stellen. Stel jouzelf daarna netjes voor, vertel naast wie je bent, bijv. ook wat je precies studeert of waarom je hieraan meewerkt. Straal een open en ontspannen houding uit, stel de deelnemer gerust. Vraag of de deelnemer wat wil drinken en haal dit voor hem/haar.  Neem daarna de deelnemer, en eventuele begeleider, mee naar de gebruikerstest ruimte. Probeer onderweg een ontspannen gesprek te creëren door wat laagdrempelige vragen te stellen, bijv.:   - Kon u het gemakkelijk vinden? - Bent u al eens eerder op de campus geweest? | Ontmoeten/ kennismaken  Oudere personen dan jijzelf standaard met ‘u’ aanspreken tenzij anders aangegeven, jongeren mag je ‘je’ zeggen. |
| *1 minuut* | Eenmaal in de betreffende ruimte, vraag de deelnemer of hij/zij al eens eerder aan zo’n gebruikersonderzoek heeft meegedaan. Leg vervolgens uit wat er binnen het onderzoek gaat gebeuren:   - Ik ga u eerst een paar vragen stellen over uzelf, de impact van corona op uw leven en uw ervaring met technologie. - Vervolgens krijgt u een telefoon van mij. Daarmee ga ik u vragen verschillende scenario’s doorlopen. Dit start met het installeren van de app, en daarna gaat u enkele stappen op de app zelf doorlopen. - Tijdens het doorlopen van deze scenario’s, vraag ik u hard op uw gedachten met ons te delen. Als u dit vergeet, probeer ik u eraan te herinneren. U kunt alles zeggen wat u denkt of voelt, of wat u ergens van vindt. De focus ligt op hoe gemakkelijk u het vindt om deze app te gebruiken en wat u van de app vindt. Het gaat er niet om hoe u de scenario’s doorloopt. U kunt niks fout doen, wij willen vooral weten wat wel en niet werkt. - Als we de scenario’s doorlopen, hebben legt u het mobieltje aan de kant en zal ik u nog een aantal vragen stellen over uw mening over de app. - Aan het einde van het onderzoek heb ik nog een korte vragenlijst die u mag invullen. - Gedurende het hele onderzoek zullen uw handelingen en gedachtes opgenomen worden via video en audio opname. De video staat gericht op het scherm van de mobiele telefoon, enkel uw handen zullen in beeld zijn. De opnamen zijn alleen voor onderzoeksdoeleinden, deze worden niet buiten ons onderzoeksteam verspreid. - Om snel de resultaten naar het VWS terug te koppelen, zal ik tussendoor wat aantekeningen maken op dit formulier (laat formulier zien). Achteraf kunnen we samen even langslopen of u vindt dat de informatie die ik op heb geschreven compleet is. Nogmaals, ik schrijf alleen op wat u van de app vindt, ik beoordeel niet hoe u het doet. | Uitleg onderzoek |
| *1 minuut* | Vraag de deelnemer of alles duidelijk is en of hij/zij nog vragen heeft. Zo ja, beantwoord deze naar behoefte. Zorg dat je met een zelfverzekerde houding antwoord. Als je iets echt niet weet, zeg je dat je dat na afloop na gaat vragen bij je collega en er nog op terugkomt. | Bevestigen dat voor de deelnemer alles duidelijk is |
| *3 minuten* | Vertel de deelnemer dat voordat jullie kunnen gaan beginnen, er schriftelijke toestemming nodig is voor deelname. Loop samen met de deelnemer het informed consent door.  Vraag nogmaals om bevestiging of alles duidelijk is, en zo ja, laat de deelnemer de informed consent ondertekenen.  Let op: als de deelnemer <16 jaar is, dan moeten ze een ondertekend informed consentformulier mee hebben genomen, dit is afgesproken. Deelnemers vanaf 16 jaar mogen zelf het informed consent ter plekke ondertekenen. | Tekenen informed consent |
| *1 minuut* | Vraag of de deelnemer klaar is om te beginnen. Als dat zo is, deel mee dat je de opnames gaat starten en start met onderstaand protocol. |  |

**1. Start: openingsvragen - Achtergrond deelnemers 🡪 max 8 minuten aanhouden**

|  | Goed, de opname is gestart. We beginnen met een kort interview waarin ik u een aantal vragen ga stellen. | |
| --- | --- | --- |
| *1 minuut* | **1. Kunt u beschrijven wat u in het dagelijkse leven zoal doet?** | Let op: informatie over school/werk/pensioen etc. Je hoeft niet alle hobby’s enz. te horen. |
| *2 minuten* | **2. Hoe groot zou je/u de impact van de corona-crisis op uw leven omschrijven op een schaal van 1 tot 10?**  Kunt u dit in een paar zinnen beschrijven? | Let op: Het doel van deze vraag is achterhalen hoeveel ‘belang’ iemand hecht aan het eindigen/oplossen van deze crisis. Als iemand heel veel impact merkt, kan het zijn dat ze ook eerder geneigd zijn om de app te gebruiken. Maar het kan een lang verhaal opleveren, dus probeer het kort te houden. Het cijfer dat ze geven, is niet zo van belang (dus is niet erg als ze daar moeite mee hebben), maar is bedoeld om de vraag wat korter te maken. |
| *2 minuten* | **3. In hoeverre heeft u angst voor corona?**   - Voor uzelf? - Voor mensen in uw omgeving? - Voor de gevolgen van de crisis? - Heeft iemand in uw omgeving (of uzelf) corona (gehad)? |  |
| *1 minuut* | **4. Bent u iemand die handig is met technologie, zoals apps en smartphones?** | Indien alleen ja/nee antwoord:   - Waar maakt u dan zoal gebruik van op uw mobiel? |
| *2 minuten* | **5. Wat heeft u tot nu toe gehoord over de Corona-apps die de overheid wil ontwikkelen?** | Opties voor doorvragen:   - Kunt u daar wat meer over vertellen? - Wat is uw gevoel hierbij? |

**2. Scenario-based usability test**

**Scenario 1 (via Figma-flow) 🡪 max. 8 minuten aanhouden**

**Installatie**

| *1 minuut* | **Vraag de deelnemer met wat voor soort mobiel hij/zij gewend is om te gaan (Android of Apple).** *Geef vervolgens het testtoestel wat het meest lijkt op hetgeen de deelnemer gewend is.*  **Stel, u heeft iets gehoord over de Corona-app van de overheid die gebruikt kan worden voor contactonderzoek. U bent nieuwsgierig en zoekt de app op in de App Store. U vindt deze informatie:**  *Laat App Store informatie zien: dit vind je via de snelkoppeling op het startscherm/in afbeeldingen (screenshots)*  <https://www.figma.com/proto/rqel74CIT7FRHurr6QoUpA/Public-Covid-19-notificatie-app-(Editable)?node-id=848%3A3955&scaling=min-zoom> | Let op: zorg dat je deze screenshots paraat hebt op laptop/tablet voor reserve. Maandag definitief besluit over hoe we deze mock-ups laten zien. |
| --- | --- | --- |
| *3 minuten* | **Wat ga je doen?** | Aandachtspunten, ook voor codeerschema:   - Lezen de deelnemers de informatie? - Vinden ze de informatie duidelijk of stellen ze er vragen over? - Gaan ze de app installeren?   Mogelijke doorvragen:   - Waarom heb je wel/niet op installeren geklikt? - Waarom ga je de app wel/niet installeren |
| *3 minuten* | Vragen na afloop scenario:   - **Zou je de app opzoeken als je er iets over gehoord had? Wanneer wel/niet?** - **Als de buurvrouw/klasgenoot zou vragen wat deze app doet, hoe zou je dat uitleggen?** | Let op: gebruik ‘buurvrouw’ bij de ouderen en ‘klasgenoot’ bij de jongeren.   - Is het een kloppend scenario, m.a.w. zouden ze al bij deze informatie in de App Store komen? - Van wie willen ze informatie ontvangen? - Welke informatie willen ze ontvangen? |

**Scenario 2 (bèta-versie van de app) – Probeer max 12-15 minuten aan te houden**

**Introductie van app en activatie van app (meldingen aanzetten)**

| **Tijd** | **Taak** | **Aandachtspunten** |
| --- | --- | --- |
| *4 minuten* | **Stel, u heeft besloten om de app te installeren. De app is net klaar met installeren en staat op het scherm. Wat gaat u nu doen?**  *Laat deelnemer de hele procedure voor 1^e^ keer gebruik doorlopen. Wanneer iemand aangeeft dat hij/zij ergens zou stoppen, doorvragen waarom, maar daarna wel verder laten gaan met het scenario.* | *Aandachtspunten:*   - *Wat vinden de deelnemers van de informatie die ze lezen?* - *Wat vinden ze van het toegang geven tot Bluetooth (let op: binnen Android moeten ze hiervoor ook toegang geven tot de locatie, terwijl dat niet in de app gebruikt wordt)* - *Doorlopen ze alle stappen volledig? Als dat niet zo is, kun je vragen: Zijn er nu nog andere dingen die je zou moeten doen voordat de app werkt?* |
| *4 minuten* | Vragen na afloop scenario (ook als het nog niet duidelijk is geworden)   - Wat vond u van de verschillende stappen die je doorlopen hebt? (Logisch, nuttig, makkelijk) - Hoe zou u nu de werking van de app uitleggen aan de buurvrouw/klasgenoot? - Kunt u uitleggen wanneer u een melding krijgt? Wat vindt u daarvan? | Let op: De app werkt globaal als volgt: telefoons met de app die >10 min. binnen een bepaalde Bluetooth-range zijn (deze range komt ongeveer overeen met 1,5 meter) wisselen een anonieme code uit. Deze code bevat geen locatie of persoonsgegevens. Wanneer iemand op een later moment een positieve testuitslag krijgt (dus is besmet met corona) en dit in de app doorgeeft, wordt een notificatie (‘u hebt risico op een corona-besmetting gelopen’) gestuurd naar de telefoons behorende bij de codes die zijn opgeslagen op de telefoon van de besmette persoon. Codes worden max 14 dagen opgeslagen, en er worden alleen notificaties gestuurd naar de codes die zijn opgeslagen in de periode dat de persoon besmettelijk was. Dit is ongeveer 2 dagen voordat de klachten begonnen. Deze informatie vraagt de GGD uit bij een positieve besmetting. In de praktijk zal dit betekenen dat mensen heel weinig meldingen zullen krijgen en dat deze meldingen gaan over contacten van een aantal dagen geleden (ongeveer tussen 2 en 7 dagen). Bij een melding is er wel degelijk verhoogde kans op een besmetting. Er wordt nooit vermeld wie positief besmet was of waar het contact heeft plaatsgevonden. Die informatie is ook niet opgeslagen. |
| *4* | *Na afloop van dit scenario zal de deelnemer automatisch op het startscherm/statusscherm uitkomen. Indien dit niet het geval is, instrueer de deelnemer het startscherm op te zoeken.*  **Stel, u heeft de app geïnstalleerd en bent op dit scherm gekomen. Wat gaat u nu doen?** | Let op: Het kan zijn dat deelnemers hier direct al gaan kijken, tijdens het scenario van het eerste gebruik. Als dat niet zo is, kun je dit voorleggen.  Ze kunnen op de verschillende onderdelen klikken, maar het is ook ok als ze zeggen: nu zou ik de app sluiten.  Mogelijke doorvragen:   - Wat verwacht u dat er gebeurt als u op de verschillende knoppen drukt? - Gebeurt dat ook? - Wat vind je daarvan? (Bijv. is het nuttig om de app te kunnen delen met anderen?) |

**Scenario 3 (bèta-versie van de app) 🡪 max 8 minuten NOTIFICATIE**

Let op!

Geef voordat je begint aan dit scenario via de app-groep aan dat je nu aan scenario 3 gaat beginnen, en dat de GGD-beller gereed moet staan. Geef hierbij het toestelnummer van de testtelefoon door (staat op achterkant/post-its).

| **Tijd** | **Taak** | **Aandachtspunten** |
| --- | --- | --- |
| *8 minuten* | **Stel, u gebruikt de app nu 10 dagen en ziet deze melding op uw telefoon:**  *Laat scherm zien met de notificatie dat je kans op besmetting hebt gelopen.*  [https://www.figma.com/proto/rqel74CIT7FRHurr6 QoUpA/Public-Covid-19-notificatie-app- (Editable)?node-id=848%3A3264&scaling =min-zoom](https://www.figma.com/proto/rqel74CIT7FRHurr6%20QoUpA/Public-Covid-19-notificatie-app-%20(Editable)?node-id=848%3A3264&scaling%20=min-zoom)  *Als je al in het prototype bent, kun je de deelnemer op de ‘wolkjes’ bovenaan het scherm laten klikken. Dan gaan ze terug naar het scherm waar je de scenario’s kan kiezen. Hier moet de deelnemers het scenario ‘een bericht ontvangen’ kiezen.*  **Wat is uw eerste reactie? Wat gaat u nu doen?** | - *Openen de deelnemers de app? Als ze dat niet doen, eerst vragen waarom niet. Daarna ze toch vragen om wel te doen (zodat we de inhoud van de informatie hierna kunnen testen).* - *Wat vinden ze van de informatie? (Maken ze zich zorgen, is het duidelijk wat er aan de hand is?)* - *Geven de deelnemers aan dat ze de GGD gaan bellen voor een test? Waarom wel/niet? Maakt het uit of je wel/geen klachten hebt? Maakt het uit als je een kwetsbare thuissituatie hebt (mensen met verhoogd risico in je gezin/familie)?* - *Wat doen ze verder? Blijven ze thuis tot ze de uitslag van de test hebben?* - *Geven de deelnemers aan dat ze de huisarts zouden bellen? Waarom wel/niet?* - Zie voor eerdere bevindingen met dit scenario: [https://corona.sticktailapp.com/share/view/ 7331484c109a484/aagGvftR6wli/de-blootstellingsmelding-voelt-nog-niet-altijd-urgent-genoeg/](https://corona.sticktailapp.com/share/view/%207331484c109a484/aagGvftR6wli/de-blootstellingsmelding-voelt-nog-niet-altijd-urgent-genoeg/) |

**Scenario 4a – met bèta-versie app 🡪 max 8 minuten. *Positief melding en gesprek GGD***

Let op: de helft van de deelnemers heeft scenario 4a, de andere helft 4b. Tot welke groep de deelnemer behoort, kun je zien in het participantnummer. In het participantnummer staat dan A of B, bijvoorbeeld Participant7A of Participant13B.

Indien scenario A: Geef nu nogmaals in de appgroep aan dat er over 2 minuten gebeld moet worden, start vervolgens met het scenario.

| Tijd | **Taak** | Aandachtspunten |
| --- | --- | --- |
|  | **Stel, u heeft u laten testen naar aanleiding van de voorgaande melding. U wordt binnen 2 dagen gebeld door iemand van de GGD met de uitslag. Toevallig weet ik dat u zo gebeld gaat worden. Vanwege dit onderzoek is dat natuurlijk niet echt, maar we willen u wel vragen om u zo goed mogelijk in te leven.**  *Telefoon gaat over. Iemand in de andere ruimte belt om te zeggen dat de deelnemer positief getest is op corona.* | - Kunnen de deelnemers de controlecode vinden? - Geven ze deze ook door?   Let op: Voor eerdere bevindingen met het testen van dit proces, zie:  <https://corona.sticktailapp.com/share/view/e1733fb74d0b64c/VC0JRwP3PCAg/aangeven-dat-je-positief-getest-bent-gaat-relatief-soepel-maar-er-is-nog-wel-verwarring-over-controlecode-en-codes-uploaden/> |
|  | Na het gesprek:   - Wat vond u hiervan? - Wat gaat u nu doen? - Als de deelnemer klikt op ‘codes uploaden’: wat gebeurt er nu denkt u? | - Lukte het om de controlecode door te geven, wilde u dat ook doen? - Wat vond u van de stappen en wat vond u het moeilijkste? - Gaat u de codes uploaden? Waarom wel/niet? - Is het duidelijk dat iedereen met wie u in contact bent tijdens uw besmettelijke periode geweest, d.w.z. >10 min in Bluetooth-range, een notificatie krijgt en wat er in die notificatie staat. |

**Scenario 4b – met bèta-app 🡪 max 8 minuten**

Indien scenario B: Geef vervolgens nogmaals aan in de appgroep dat er gebeld moet worden door de GGD binnen # minuten. Maak zelf een schatting in aantal minuten, afhankelijk van het leestempo van deelnemer (eerst flyer doorlezen).

| 1 | **Stel, je hebt je laten testen naar aanleiding van de voorgaande melding. Bij het testen heb je deze flyer meegekregen:**  *Geef de deelnemer de flyer. Ze mogen het lezen, maar het hoeft niet.* | *Aandachtspunten:*   - *Wat vinden de deelnemers van de informatie?* - *Lezen ze de informatie?* |
| --- | --- | --- |
|  | **Je wordt dus binnen 2 dagen gebeld door iemand van de GGD met de uitslag. We weten toevallig dat je zo gebeld wordt. Vanwege dit onderzoek is dat natuurlijk niet echt, maar we willen je wel vragen om je zo goed mogelijk in te leven.**  *Telefoon gaat over. Iemand in de andere ruimte belt om te zeggen dat de deelnemer positief getest is op corona.* | Aandachtspunten:   - Kunnen de deelnemers de controlecode vinden? - Geven ze deze ook door?   Let op: Voor eerdere bevindingen met het testen van dit proces, zie:  <https://corona.sticktailapp.com/share/view/e1733fb74d0b64c/VC0JRwP3PCAg/aangeven-dat-je-positief-getest-bent-gaat-relatief-soepel-maar-er-is-nog-wel-verwarring-over-controlecode-en-codes-uploaden/> |
|  | Na het gesprek:   - **Wat vond u hiervan?** - **Wat gaat u nu doen** - **Als de deelnemer klikt op ‘codes uploaden’: wat gebeurt er nu denkt u?** | *Aandachtspunten:*   - Lukte het om de controlecode door te geven, wilde u dat ook doen? - Wat vond u van de stappen en wat vond u het moeilijkste? - Gaat u de codes uploaden? Waarom wel/niet? - Is het duidelijk dat iedereen met wie u in contact bent tijdens uw besmettelijke periode geweest, d.w.z. >10 min in Bluetooth-range, een notificatie krijgt en wat er in die notificatie staat? |

**3. Afsluitend interview + korte vragenlijst – max 10 minuten**

| Dat was het laatste onderdeel van de gebruikerstest. U mag het toestel weer neerleggen. Graag wil ik u nog wat vragen stellen over wat u van de app vond. | |
| --- | --- |
| 1. Nu je de app hebt gezien, wat vind je hiervan?  Wil je de app gebruiken? Waarom wel/niet?  Welke twijfels heb je? | *Eventueel doorvragen, als het nog niet duidelijk is:*   - *Zou men zich voor de app aanmelden* - *Zou men app gebruiken (o.a. Notificaties in de gaten houden, lezen etc.)* - *Gaat men de adviezen opvolgen (t.a.v. testen, quarantaine etc.)* - *Gaat men testresultaat in app zetten (wanneer wel/ niet)* |
| 2. We hebben u eerder gevraagd om de app uit te leggen aan uw buurvrouw/klasgenoot. Hoe zou u dat nu doen, nu u de app daadwerkelijk hebt ervaren? |  |
| 3. Wat vindt u goed aan de app/wat spreekt u aan? |  |
| 4. Wat vindt u minder goed? | *Denken ze bijvoorbeeld dat er ook negatieve effecten zijn van het gebruik van de app?* |
| 5.Wat zou er nog duidelijker kunnen?   - Welke verbeterpunten heb je over hoe de app is vormgegeven? - Voor de inhoud? |  |
| Als u een cijfer tussen de 1 en 10 zou moeten geven voor de app. Welk rapportcijfer zou u de app geven? |  |
| Denk je dat mensen uit je omgeving de app zouden gaan gebruiken?  Waarom wel/niet? |  |

Rond het onderzoek af, deel mee dat je de opname gaat stoppen. Loop samen met de deelnemer het ingevulde beoordelingsformulier langs, vraag of hij/zij het eens is met wat je hebt opgeschreven of nog aanvullingen heeft.

Geef vervolgens de korte vragenlijst aan de deelnemer en vraag of hij/zij deze kan invullen. Terwijl de deelnemer de vragenlijst invult, kun je alvast de voorbereidingen treffen voor de volgende deelnemer (zie voorbereiding op eerste pagina; app opnieuw installeren, toestel schoonmaken etc.).

Na het invullen van de vragenlijst: bedank deelnemer vriendelijk voor deelname en overhandig cadeaubon. Loop samen met hem/haar naar de uitgang. Als je alles in de ruimte hebt voorbereid voor de volgende deelnemer, kun je deze tegelijkertijd op de terugweg mee nemen.
