# Supplementary material for: The Dutch COVID-19 Contact Tracing App (the CoronaMelder): Usability Study
Source: JMIR Form Res. 2021 Mar 26;5(3):e27882. doi: 10.2196/27882 (PMC8006901; doi:10.2196/27882)
Supplement: Multimedia Appendix 2 [file formative_v5i3e27882_app2.docx]

## Appendix 2 – PHA Telephone script for positive test results

| **GGD-medewerker** | **Respondent** |
| --- | --- |
| Goedendag, Mijn naam is Job/Lia. Ik werk bij de GGD, afdeling infectieziekten. Ik bel vanwege corona. Spreek ik met [naam respondent]? |  |
| Er is twee dagen geleden een Coronatest bij u afgenomen. We bellen u met de uitslag. Deze uitslag is helaas positief. |  |
| Heeft u op dit moment klachten? |  |
| Normaal zouden we nu met u door de richtlijnen heen lopen. Vanwege dit onderzoek slaan we deze stap over.  [De richtlijnen wanneer u positief bent getest, zijn:  ● Blijf thuis totdat de testuitslag bekend is, ontvang geen bezoek.  ● Blijf zoveel mogelijk uit de buurt van je huisgenoten  ● Zorg voor goede persoonlijke hygiëne  ● Zorg voor een goede hygiëne in uw kamer en in huis  ● Zorg voor een goede hygiëne met bevuilde spullen  Deze richtlijnen worden ook per email naar u verstuurd.] |  |
| We gaan beginnen met het bron- en contactonderzoek. Daarvoor zouden we graag willen weten of u de Corona-app heeft geïnstalleerd? |  |
| Fijn! U kunt in de app de personen die dicht bij u in de buurt geweest zijn waarschuwen. Dit gaan we samendoen terwijl we aan de telefoon zitten. We zouden u willen vragen om uw app te openen om uw data te uploaden. (Via ‘ik ben positief getest’ en ‘data uploaden’) | Respondent opent de app, gaat naar ‘heb je corona?’ en klikt dan op ‘data uploaden’. Dan ‘toestemming geven’ |
| Als het goed is, ziet u nu een code op uw scherm. Zou u deze code aan mij door kunnen geven? |  |
| Dit klopt, bedankt. U kunt nu verder gaan naar uploaden om mensen met wie u in contact bent geweest te waarschuwen. |  |
| We gaan u later vandaag terugbellen voor het bron- en contactonderzoek en verdere adviezen. |  |
| Beterschap, hopelijk voelt u zich snel weer beter! |  |
