# Supplementary material for: The Dutch COVID-19 Contact Tracing App (the CoronaMelder): Usability Study
Source: JMIR Form Res. 2021 Mar 26;5(3):e27882. doi: 10.2196/27882 (PMC8006901; doi:10.2196/27882)
Supplement: Multimedia Appendix 3 [file formative_v5i3e27882_app3.docx]

## Appendix 3 – Questionnaire (UEQ-Dutch)

# **Vragenlijst voor deelnemers gebruikerstesten Corona-app**

1. **Ik ben een…**

- Man
- Vrouw
- …
- Wil ik niet zeggen

1. **Hoe oud ben je?**

- ____ jaar
- Wil ik niet zeggen

1. **Wat is de hoogste opleiding die je hebt afgemaakt?**

- Geen of basisonderwijs
- LBO/ VMBO (kader- of beroepsgericht)/ MBO 1/ VBO
- VMBO (theoretisch of gemengd)/ MAVO/ (M)ULO/ HAVO of VWO (overgegaan naar 4e klas)
- MBO 2, 3, 4 of MBO vóór 1998
- HAVO of VWO (met diploma afgerond)/ HBS/ MMS
- Propedeuse, HBO of universitair niveau
- Bachelor/kandidaats (HBO of universitair niveau)
- Master/doctoraal/postdoctoraal (HBO of universitair niveau)

1. **Zijn er dingen waardoor je soms moeite hebt om een app op je telefoon te gebruiken?** *Meerdere antwoorden mogelijk*

- Ik heb moeite met lezen
- Ik ben dyslectisch
- Ik ben slechtziend
- Ik heb een motorische beperking
- Ik ben slechthorend
- Ik ben niet handig met digitale apparaten
- Anders, namelijk…
- Nee

1. **Hoe handig vind jij jezelf bij het gebruiken van digitale producten zoals computers, telefoons en apps?**

- Zeer handig
- Handig
- Niet handig, niet onhandig
- Onhandig
- Zeer onhandig
- Weet ik niet, geen mening

**Maak dan nu uw evaluatie.**

Voor de beoordeling van het product, vragen we u de onderstaande vragenlijst in te vullen. De vragenlijst bestaat uit twee tegengestelde eigenschappen die van toepassing zijn op het product. De rondjes staan voor verschillende gradaties. U kunt uw beoordeling geven door het rondje, die het meest uw indruk weerspiegelt, aan te vinken.

Voorbeeld:


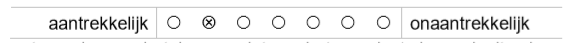
:

Graag uw eerste ingeving invullen. Wacht niet te lang met invullen om te voorkomen dat u gaat twijfelen over uw eerste ingeving. Soms bent u misschien niet helemaal zeker van uw antwoord of u vindt de eigenschap niet volledig van toepassing, kruis dan toch een rondje aan. Het is uw mening die telt. Let op: er is geen goed of fout antwoord!


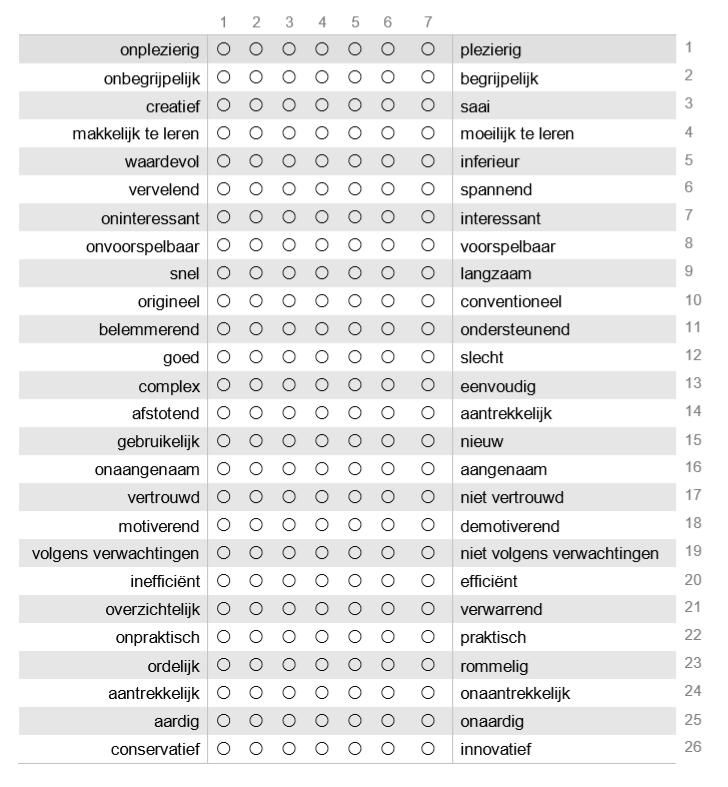
 Gelieve het product nu te beoordelen door het aanvinken van een rondje per regel.
