# Supplementary material for: The Dutch COVID-19 Contact Tracing App (the CoronaMelder): Usability Study
Source: JMIR Form Res. 2021 Mar 26;5(3):e27882. doi: 10.2196/27882 (PMC8006901; doi:10.2196/27882)
Supplement: Multimedia Appendix 4 [file formative_v5i3e27882_app4.docx]

## Appendix 4.

|  |  |  | Positive | Negative |
| --- | --- | --- | --- | --- |
|  |  |  | Do understand | Do not understand |
|  |  |  |  |  |
| **User-friendliness** | **Lay-out** |  | 23 (53%) | 8 (18%) |
|  |  | Young people, lower level of education | 8 (57%) | 5 (36%) |
|  |  | Young people, higher level of education | 5 (100%) | - |
|  |  | Young people, with an intellectual disability | 1 (25%) | - |
|  |  | Migrants | - | 1 (50%) |
|  |  | Adults | 3 (60%) | 1 (20%) |
|  |  | Elderly | 6 (43%) | 1 (7%) |
|  | **Navigation** |  | 33 (75%) | 9 (20%) |
|  |  | Young people, lower level of education | 12 (86%) | 1 (7%) |
|  |  | Young people, higher level of education | 5 (100%) | 1 (20%) |
|  |  | Young people, with an intellectual disability | 2 (50%) | - |
|  |  | Adults | 4 (80%) | 3 (60%) |
|  |  | Elderly | 10 (71%) | 4 (29%) |
| **Understandability** | **Language** |  | 7 (16%) | 10 (23%) |
|  |  | Young people, lower level of education | 1 (7%) | 3 (21%) |
|  |  | Young people, higher level of education | 2 (40%) | - |
|  |  | Young people, with an intellectual disability | 1 (25%) | - |
|  |  | Migrants | - | 2 (100%) |
|  |  | Adults | 1 (20%) | - |
|  |  | Elderly | 2 (14%) | 5 (36%) |
|  | **Receiving a notification** |  | 15 (34%) | 21 (48%) |
|  |  | Young people, lower level of education | 3 (21%) | 9 (64%) |
|  |  | Young people, higher level of education | 3 (60%) | 1 (20%) |
|  |  | Young people, with an intellectual disability | - | 2 (50%) |
|  |  | Migrants | - | 1 (50%) |
|  |  | Adults | 1 (20%) | 3 (60%) |
|  |  | Elderly | 7 (50%) | 5 (36%) |
|  | **Sharing the key** |  | 15 (34%) | 19 (43%) |
|  |  | Young people, lower level of education | 6 (43%) | 5 (36%) |
|  |  | Young people, higher level of education | 2 (40%) | 2 (40%) |
|  |  | Young people, with an intellectual disability | - | 4 (100%) |
|  |  | Migrants | 1 (50%) | 1 (50%) |
|  |  | Adults | 2 (40%) | 1 (20%) |
|  |  | Elderly | 4 (29%) | 6 (43%) |
| **Reliability and credibility** |  |  | 19 (43%) | 6 (14%) |
|  |  | Young people, lower level of education | 4 (29%) | 3 (21%) |
|  |  | Young people, higher level of education | 3 (60%) | - |
|  |  | Young people, with an intellectual disability | 1 (25%) | - |
|  |  | Adults | 3 (60%) | 1 (20%) |
|  |  | Elderly | 8 (57%) | 2 ((14%) |
| **Inclusiveness** |  |  | 5 (11%) | 9 (20%) |
|  |  | Young people, lower level of education | 3 (21%) | 1 (7%) |
|  |  | Young people, higher level of education | - | - |
|  |  | Young people, with an intellectual disability | - | 1 (25%) |
|  |  | Adults | - | 1 (20%) |
|  |  | Elderly | 2 (14%) | 6 (43%) |

Appendix 4. Number of participants per target group who stated a positive or negative comment about the CoronaMelder, per topic (user-friendliness, understandability, reliability & credibility, and inclusiveness). In case of the understandability of the notification and sharing the key, the table shows how many participants understood the working of the CoronaMelder app.
