# Supplementary material for: The Dutch COVID-19 Contact Tracing App (the CoronaMelder): Usability Study
Source: JMIR Form Res. 2021 Mar 26;5(3):e27882. doi: 10.2196/27882 (PMC8006901; doi:10.2196/27882)
Supplement: Multimedia Appendix 5 [file formative_v5i3e27882_app5.docx]

*
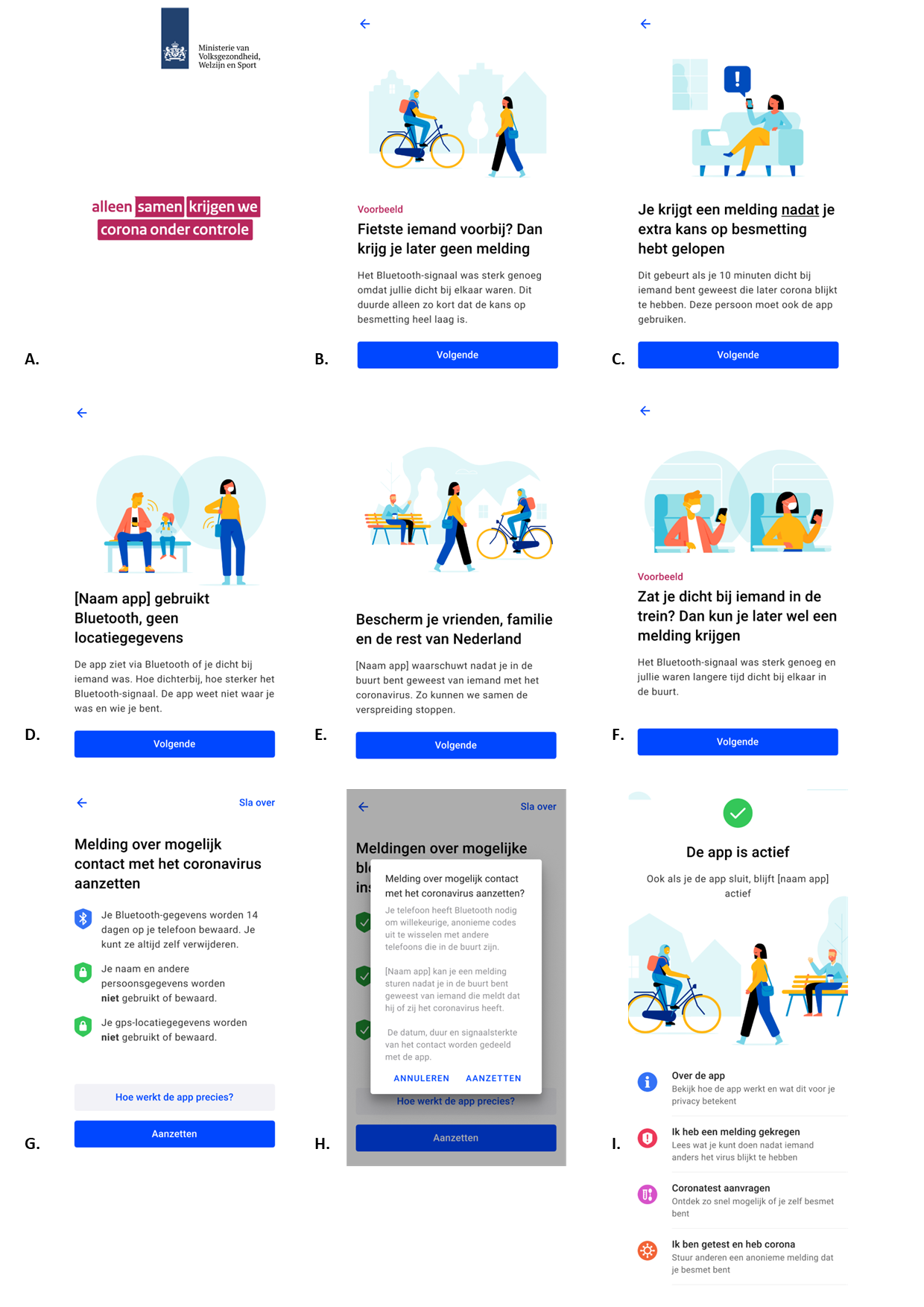
*

Appendix 5. Figures A-I show screenshots of onboarding and activation of the app. The screenshots include the following information: A) Start screen with Dutch COVID-19 combat slogan; B) How the app operates; C) When will you receive a notification; D) Explanation on how the app uses Bluetooth and no additional data; E) Example of when you will not receive a notification (Figure 2); F) Example of when you will receive a notification (Figure 3); G), Giving permission to allow the app to use Bluetooth; H), Giving permission to receive notifications from the app; I) The app’s home screen, which appears after the onboarding steps are performed and the app is activated and ready for use (Figure 5).
